# SURGICAL DELAY IN PATIENTS WITH SUBAXIAL FRACTURE

**DOI:** 10.1590/1413-785220253303e289304

**Published:** 2025-08-18

**Authors:** Deusimar Cristian dos Santos Gomez, Fabrício Luz Cardoso, Gabriel José dos Santos, Robert Meves, Maria Fernanda Silber Caffaro, Jefferson Walter Daniel

**Affiliations:** 1 Irmandade Santa Casa de Misericordia de São Paulo (ISCMSP), Departamento de Ortopedia e Traumatologia, São Paulo, SP, Brazil.; 2 Irmandade Santa Casa de Misericordia de São Paulo (ISCMSP), Departamento de Neurocirurgia, São Paulo, SP, Brazil.

**Keywords:** Cervical Vertebrae, Spinal Cord Injuries, Surgical Procedures, Operative, Fractures, Bone, Vértebras Cervicais, Traumatismos da Medula Espinal, Procedimentos Cirúrgicos Operatórios, Fraturas Ósseas

## Abstract

**Objective:**

This retrospective study investigated the waiting time for surgery in patients with severe subaxial cervical fractures at Santa Casa de Misericórdia de São Paulo, as well as identifying the main causes of surgical delays.

**Methods:**

The research was quantitative and retrospective, utilizing medical records of patients operated on between January 2015 and June 2023. Variables analyzed included age, gender, trauma mechanism, fracture classification, neurological status, waiting time until surgery, and causes of delay. Data were initially collected using physical forms and then migrated to electronic platforms (SurveyMonkey^®^ and Red Cap^®^) for detailed statistical analysis.

**Results:**

The study included 36 patients, with a significant predominance of men (86.1%) and an average age of 44.97 years. Falls were the most common trauma mechanism (44.4%), followed by automobile accidents (27.8%). Most fractures occurred at the C4/C5 (16.7%) and C5/C6 (13.9%) levels. The most frequent AO type classification was type C (47.2%). The average waiting time for surgery was 9.28 days, with the main cause of delay being the availability of the operating room (66.7%).

**Conclusion:**

The results indicate that cervical fractures have a significant impact on young adults, predominantly men, often associated with falls and automobile accidents. Early identification and timely surgical intervention are crucial to minimize complications and improve neurological outcomes. Strategies to reduce surgical waiting times, such as improvements in hospital resource management, are essential to optimize the treatment of these injuries. ***Level of Evidence III; Retrospective Cohort Study.***

## RESUMO

**Objetivo:**

este estudo retrospectivo investigou o tempo de espera para cirurgia em pacientes com fraturas cervicais subaxiais graves na Santa Casa de Misericórdia de São Paulo, além de identificar as principais causas de atraso nas cirurgias.

**Métodos:**

a pesquisa foi quantitativa e retrospectiva, utilizando dados de prontuários médicos de pacientes operados entre janeiro de 2015 a junho de 2023. Foram analisadas variáveis como idade, gênero, mecanismo do trauma, classificação da fratura, estado neurológico, tempo de espera até a cirurgia, e causas de atraso. Os dados foram coletados inicialmente em formulário físico e depois migrados para plataformas eletrônicas (SurveyMonkey^®^ e Red Cap^®^) para análise estatística detalhada.

**Resultados:**

o estudo incluiu 36 pacientes, com predominância significativa de homens (86,1%) e idade média de 44,97 anos. Quedas foram o mecanismo de trauma mais comum (44,4%), seguidas por acidentes automobilísticos (27,8%). A maioria das fraturas ocorreu nos níveis C4/C5 (16,7%) e C5/C6 (13,9%). A classificação AO tipo C foi a mais frequente (47,2%). A média de tempo de espera para cirurgia foi de 9,28 dias, com a principal causa de atraso sendo a disponibilidade de sala cirúrgica (66,7%).

**Conclusão:**

os resultados indicam que as fraturas cervicais têm um impacto significativo em adultos jovens, principalmente homens, frequentemente associadas a quedas e acidentes automobilísticos. A identificação precoce e a intervenção cirúrgica oportuna são cruciais para minimizar complicações e melhorar os resultados neurológicos. Estratégias para reduzir o tempo de espera cirúrgica, como melhorias na gestão hospitalar de recursos, são essenciais para otimizar o tratamento dessas lesões. ***Nível de Evidência III; Estudo de Coorte Retrospectivo.***

## INTRODUCTION

Subaxial cervical spine fractures (C3 to C7) are common and often require surgical intervention due to their severity.^1^ The Allen and Ferguson classification describes these lesions according to the mechanisms of trauma, while the AO classification divides the fractures into categories based on the mechanism of injury (A - compression, B - distraction, C - rotation).^2,3^

Spinal Cord Injury (SCI) compromises the bone, ligament, joint, muscular, vascular or neural structures of the spine as a result of a direct or indirect physical event.^4^ Accurate information on the prevalence and incidence of SCI at national level is scarce.

Epidemiological data are predominantly derived from regional studies.^5^ Global estimates suggest that SCI prevalence ranges from 236 to 4187 cases per million inhabitants.^5^ In Brazil, it is estimated that 6 to 8 thousand new cases of SCI occur annually. In the United States, the incidence is estimated at 13,000 patients per year, with considerable costs for the health and social security system ranging from $1.1 to $4.6 million per patient.^6^

One study examined 160.455 cases of cervical fractures over 10 years, showing a higher incidence of fractures in the sub-axial cervical spine (65% of cases) compared to the upper cervical spine (35%).^7^ The prevalence of SCI is considerably higher in men, about 3 to 4 times higher than in women.^8^

Subaxial cervical trauma is evaluated independently from other regions of the spine. Vaccaro et al.^6^ developed the classification system SLICS (Subaxial Cervical Spine Injury Classification System), which considers three main aspects: fracture morphology, integrity of the posterior ligament complex and the patient's neurological status.^9^ The overall score is obtained by summing the individual scores of each aspect. The higher the score, the more severe the injury, according to the SLICS system.

The posterior ligamentous complex encompasses structures at the back of the vertebral column, including the zygapophyseal joints, ligamentum flavum, and interspinous ligaments. Injuries to this complex indicate spinal instability and may result in subluxations or dislocations of the facet joints.^10^ Injuries with less than 4 points are considered mechanically stable and generally do not require surgical intervention, while injuries with more than 4 points are considered unstable and require surgery.

Patients with SLICS score below 4 points should be monitored with periodic X-rays to monitor cervical alignment during the fracture consolidation process, as well as clinical evaluations. Monitoring may be suspended when signs of complete consolidation of the fracture are observed, but it is important to be aware of indicators of instability, such as body height loss or increased fracture angle and the presence of listesis.^11^

If the patient is clinically stable and without neurological impairment, a MRI is recommended prior to surgery to identify hidden disc hernia or compressions due to degenerative lesions.^12^

The application of cranial traction is appropriate for patients with cervical misalignments resulting from facet dislocation, provided they are conscious, have a reliable neurological examination, and are not candidates for immediate surgery. During the traction process, the load is gradually increased with appropriate analgesia, while lateral cervical X-rays are obtained to visualize the vertebrae from C3 to T1. This technique provides a rapid decompression of neural tissue and can be used as a temporary measure until the final surgical procedure is performed.^13^

Factors associated with surgical delay include the availability of hospital resources, the clinical stability of the patient, the need for additional diagnostic investigations and the complexity of the case. Rapid identification and treatment of lesions in the subaxial cervical spine is crucial for maximizing chances of recovery and reducing complications.

This study aims to describe the waiting time for surgery in patients with cervical fractures type A3, A4, B and C at the largest philanthropic hospital in Latin America located in the city of São Paulo and identify the main causes of delays in surgeries.

## METHODOLOGY

This retrospective study is a quantitative research focused on the analysis of epidemiological data extracted from medical records and the comparison with current literature.

Inclusion criteria: A3 and A4, B or C fractures according to AO Spine classification; Patients operated between January 2015 and June 2023 in the department of orthopaedia and traumatology or neurosurgery of the largest philanthropic hospital in Latin America located in the city of São Paulo; Ages between 18 and 80; Availability of detailed clinical history in the medical record.

Exclusion criteria: Fractures type A0, A1, A2; fractures type B and C not surgically treated; incomplete data.

The study was approved by the Ethics and Research Committee of the Arnaldo Vieira de Carvalho Foundation (CAAE: 76159023.4.0000.5479).

**Figure 1:**
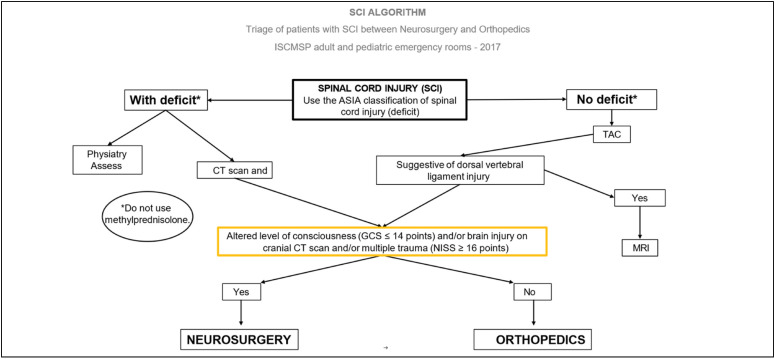
SCI algorithm.

The patients of the institution are sorted in the Red Room (trauma room) by the General Surgery team, following the Spinal Cord Injury – SCI algorithm (Figure 1). This screening process aims to determine, in collaboration with the specialties of neurosurgery and orthopedics, which one will be responsible for the clinical and/or surgical follow-up of the patient.

The data collection was carried out in three stages. The first step involved extracting information from medical records using a physical form (Appendix 1), including details such as age, gender, mechanism of trauma, comorbidities, hospitalization time, level of injury, fracture classification, neurological status, American Spinal Injury Association score, number of instrumented levels, and details of surgical intervention.

In the second step, the data was converted to electronic format using the SurveyMonkey^®^ and Red Cap^®^ platforms. SurveyMonkey^®^ facilitated the inclusion of data in the multicenter study promoted by AO Spine, while Red Cap^®^ was used to organize and process the data, ensuring efficiency and accuracy in the analysis.

In the third step, the statistical analysis was carried out with the Statistica^®^ software. Various statistical tests were applied, including t-Student, Pearson correlation coefficient, Mann-Whitney U, qui-square, Spearman correlation, Shapiro-Wilk normality test, variance analysis (ANOVA) and Levene, to evaluate the collected data and identify significant patterns.

## RESULTS

Table 1 presents the results of the variables observed in the study. The gender distribution revealed a significant majority of men, totaling 31 participants, which represents approximately 86.1% of the sample. In contrast, the female presence in the sample was substantially lower, with only 5 female participants, corresponding to about 13.9% of the total. The standard gender deviation was estimated at approximately 0.349.

**Table 1:** Details of selected cases of orthopedics and neurosurgery.

Gender	Age	Comorbidity	Trauma	Arrival at the Hospital	Fracture Level	Classification AO Spine	ASIA	Glasgow scale on admission	NISS	Date of surgery	Admission to surgical period	Instrumentation Level	Complications	Days of internment	Motive of surgical delay	Specialty
Male	38	Without Comorbidities	Motorcycle	10/11/2016	C6/C7	C	E	15		10/31/2016	20	C6-C7	No complications	25	Unavailability of Room	Orthopedics
Male	42	Hypertension	Fall	05/08/2017	C5/C6	A4	C	12	24	05/09/2017	1	C3-C6	No complications	17	Unavailability of Room	Neurosurgery
Male	46	Hypertension	Motorcycle	06/09/2017	C5/C6/C7	B1	D	14	14	06/21/2017	12	C4-T1	No complications	19	Clinical Instability	Neurosurgery
Female	50	Hypertension	Car	08/01/2017	C6/C7	B2	C	15	14	08/02/2017	1	C4-C7	No complications	25	Unavailability of Room	Neurosurgery
Male	30	Without Comorbidities	Others	08/09/2017	C6	A4	C	15		08/16/2017	7	C5-C7	No complications	11	Unavailability of UTI Bed	Orthopedics
Male	48	Without Comorbidities	Motorcycle	09/16/2017	C3	A4	C	3	24	10/03/2017	17	C3-C4	Pneumonia	60	Clinical Instability	Neurosurgery
Male	71	Without Comorbidities	Fall	01/01/2018	C5	A3	D	15		01/08/2018	7	C3-C6	No complications	32	Unavailability of Room	Orthopedics
Male	60	Without Comorbidities	Fall	01/21/2018	C3	A3	B	15		01/29/2018	8	C3-C6	Pulmonary Embolism / Surgical Infection	239	Unavailability of Room	Orthopedics
Male	58	Without Comorbidities	Others	02/06/2018	C5/C6	C	E	15		02/26/2018	20	C4-C6	No complications	39	Unavailability of Room	Orthopedics
Male	58	Without Comorbidities	Others	02/08/2018	C4/C5	C	D	15		02/14/2018	6	C4-C5	Postoperative neurological deterioration	175	No delay	Orthopedics
Male	63	Without Comorbidities	Fall	03/07/2018	C5	A3	A	15		03/12/2018	5	C3-T1	Death	8	Unavailability of Room	Orthopedics
Female	31	Without Comorbidities	Others	08/21/2018	C4/C5	C	C	15		08/27/2018	6	C3-C4	No complications	141	Unavailability of Room	Orthopedics
Male	37	Without Comorbidities	Others	10/06/2018	C6	C	A	15		10/15/2018	9	C3-C4	Death	109	Unavailability of Room	Orthopedics
Male	57	Without Comorbidities	Fall	10/31/2018	C4/C5	C	A	15		11/07/2018	7	C4-C5	No complications	13	Unavailability of Room	Orthopedics
Male	63	Hypertension	Fall	07/30/2019	C5/C6/C7	B1	D	15	17	07/31/2019	1	C4-C7	No complications	4	Unavailability of Room	Neurosurgery
Male	40	Lung diseases	Others	08/20/2019	C7/T1	A4	D	3	29	10/08/2019	49	C6-T2	Death	72	Clinical Instability	Neurosurgery
Male	30	Without Comorbidities	Car	09/13/2019	C5	A4	C	15	18	09/25/2019	12	C4-C6	Urinary Infection	5	Clinical Instability	Neurosurgery
Male	48	Hypertension	Fall	09/28/2019	C4/C5	B2	D	15	18	10/03/2019	5	C3-C6	No complications	10	Unavailability of Implants	Neurosurgery
Male	80	Hypertension	Fall	12/30/2019	C3/C4	C	A	15		01/06/2020	6	C3-T1	No complications	28	Unavailability of Room	Orthopedics
Male	57	Others	Fall	01/11/2020	C3/C4	B1	C	14	24	01/14/2020	3	C3-C4	Death	140	Unavailability of Implants	Neurosurgery
Male	29	Without Comorbidities	Fall	01/26/2020	C5	C	B	14	11	02/03/2020	8	C3-C7	Postoperative neurological deterioration	30	Unavailability of Implants	Neurosurgery
Male	37	Without Comorbidities	Dive	02/09/2020	C5	B2	A	15		02/12/2020	3	C3-C7	Deiscence of Operational Wound	102	Unavailability of Implants	Orthopedics
Male	55	Without Comorbidities	Fall	03/30/2020	C7/T1	C	C	14	16	04/01/2020	2	C5-C6-T1-T2	No complications	24	Unavailability of Room	Neurosurgery
Male	49	Smoking	Fall	04/04/2020	C4/C5	C	C	15		04/13/2020	9	C4-C5	No complications	13	Unavailability of Room	Orthopedics
Male	38	Without Comorbidities	Car	05/31/2020	C3/C4	C	A	15		06/08/2020	8	C3-C6	Death	47	Unavailability of Room	Orthopedics
Male	22	Without Comorbidities	Motorcycle	06/01/2020	C1/C6	C	E	15	18	06/04/2020	3	C5-C7	No complications	6	Unavailability of Implants	Neurosurgery
Male	64	Smoking / Lung Diseases	Fall	10/14/2020	C7	B2	E	15		10/28/2020	14	C6-C7	No complications	16	Unavailability of Room	Orthopedics
Male	20	Without Comorbidities	Motorcycle	06/18/2021	C6	C	A	15	16	06/22/2021	4	C5-C7	Pneumonia	25	Unavailability of Implants	Neurosurgery
Male	81	Diabetes	Fall	10/08/2021	C5/C6	B3	A	15		10/18/2021	10	C3-T1	Death	13	Unavailability of Room	Orthopedics
Female	41	Without Comorbidities	Dive	01/15/2022	C6/C7	B2	E	15		01/24/2022	9	C6-T1	No complications	13	Unavailability of Room	Orthopedics
Male	28	Without Comorbidities	Dive	03/01/2022	C7	A4	D	15		03/14/2022	13	C5-T1	No complications	17	Unavailability of Room	Orthopedics
Male	39	Ethylism / Smoking	Motorcycle	03/03/2022	C6/C7	C	E	15		04/04/2022	31	C6-C7	No complications	34	Unavailability of Room	Orthopedics
Female	74	Ethylism	Fall	03/09/2022	C6	C	A	14	16	03/11/2022	2	C5-C7	Pneumonia / death	97	Unavailability of Room	Neurosurgery
Female	83	Hypertension	Fall	05/29/2022	C5/C6	B2	B	15		06/20/2022	22	C3-C7	No complications	34	Unavailability of Room	Orthopedics
Male	25	Without Comorbidities	Dive	12/12/2022	C7/T1	C	B	15		12/19/2022	7	C7-T1	No complications	10	Unavailability of Room	Orthopedics
Male	31	Without Comorbidities	Others	01/21/2023	C4/C5	C	A	15		01/24/2023	3	C4-C5	No complications	8	Unavailability of Room	Orthopedics

The general average of ages is approximately 44.97 years and the standard deviation is approximately 18.7 years.

We observed that most were treated by orthopaedics, representing approximately 61.1% of the cases (22 patients). On the other hand, neurosurgery provided assistance to about 38.9% of the cases (14 patients).

**Figure 2:**
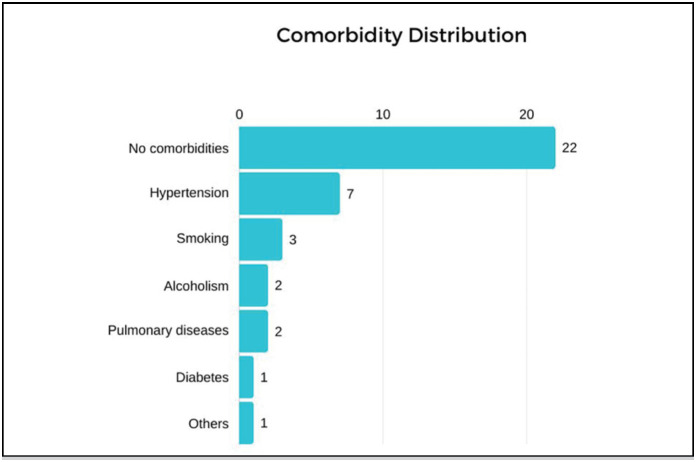
Distribution of comorbidities.

Of the 36 participants included in the study, the majority (22) did not report any registered morbidity, representing 61.1% of the total, the distribution of these comorbidities is represented in Figure 2.

**Figure 3:**
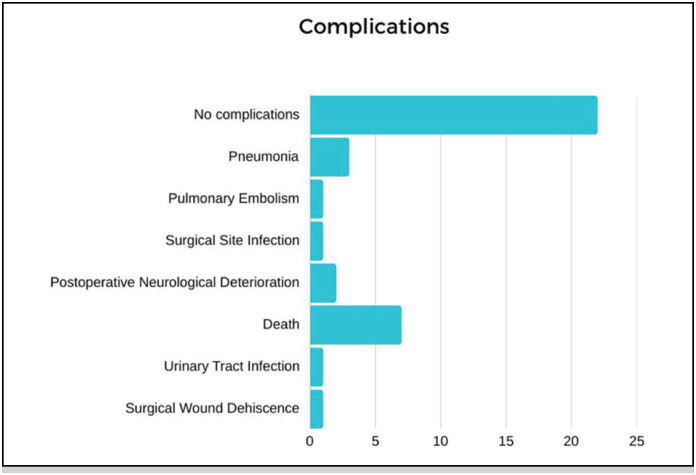
Types of trauma.

Various types of trauma as represented in Figure 3 have been described. Falls were the most common cause, affecting 16 participants.

**Figure 4:**
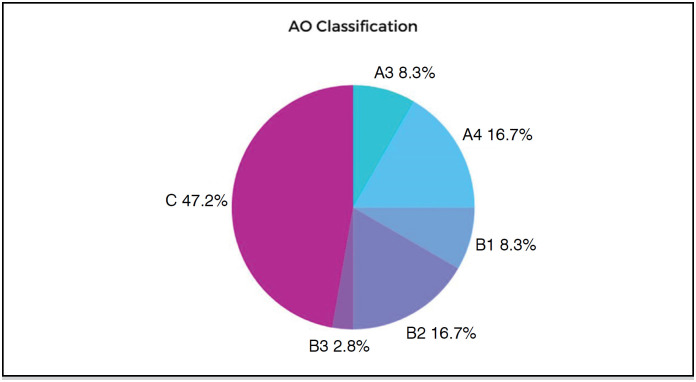
Levels of fractures.

Several cervical fractures have been recorded. The most common level of fracture was C4/C5 affecting 6 patients. Figure 4 shows this distribution.

**Figure 5:**
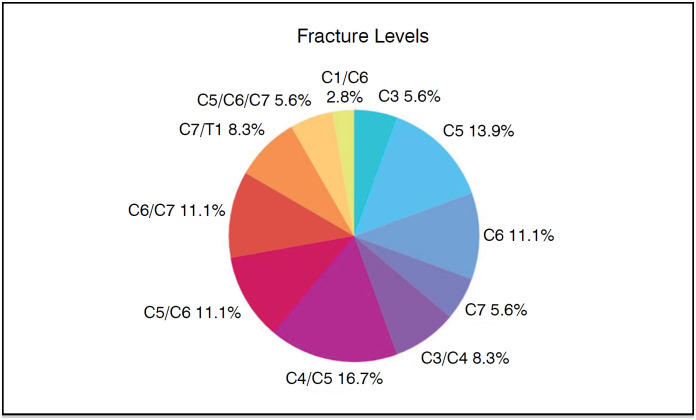
Classification AO.

The fractures were classified according to the AO Spine Classification. The most common classification was type C, identified in 17 participants, which corresponds to approximately 47.2% of the total. The distribution of the classification is shown in Figure 5.

The injuries were classified according to the American Spinal Injury Association (ASIA) Medular Injury Scale. The most frequent category was A, identified in 10 participants, representing 27.78% of the total. Category C followed with 9 participants (25.00%), while category D was observed in 7 participants (19.44%). Categories B and E were less common, with 5 participants each (13.89%).

At admission, patients are sorted by the Glasgow Scale. The most frequent score was 15, recorded in 28 participants (77.78%). Score 14 appeared in 5 participants (13.89%). Scores 3 and 12 were less common, with 2 participants (5.56%) and 1 participant (2.78%), respectively.

Analysis of the interval between admission and surgical procedure revealed an average of approximately 9.28 days, with a standard deviation of 9.58 days. About 61.11% of patients were operated in less than 9.28 days after admission. Approximately 30.56% underwent the procedure between 9.28 and 18.86 days, while 8.33% were operated after 18.86 days.

As for the level of instrumentation, most of the 36 participants received instrumentation at levels C3-C6 and C3-C4 (5 patients each, 13.89% each). C5-C7 and C4-C5 levels were frequent, with 4 patients each (11.11% each). Other significant levels were C4-C7, C6-C7, C3-T1, C4-C6 and C3-C7 (3 patients each, 8.33%). Levels such as C4-T1, C6-T2, C5-C6-T1-T2, C6-T1, C5-T1 and C7-T1 were less common, with 1 patient each (2.78%).

**Figure 6:**
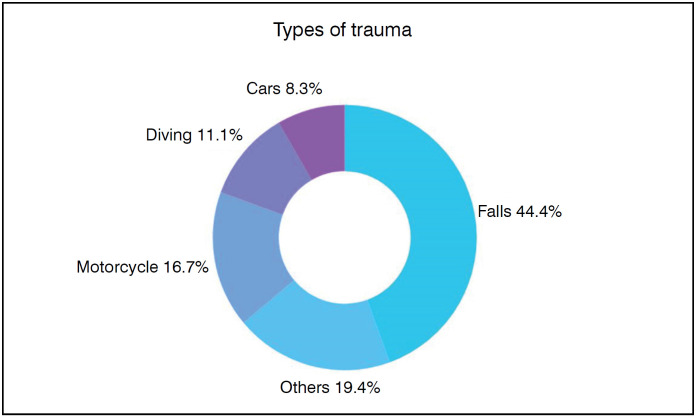
Complications.

Figure 6 represents the complications found in the hospitalizations of study participants. The majority of participants 22 (61.1%) did not experience any complications during their hospitalization.

The analysis of hospitalization data revealed an average of approximately 45.33 days, with a standard deviation of 54.38 days. Dividing patients into three hospitalization time ranges, it was observed that none of the participants had a stay below the average minus a standard deviation.

Among the patients, 30 (83.33%) were hospitalized for a period between the average minus one standard deviation and the average plus one standard deviation. Finally, 6 patients (16.67%) remained hospitalized for longer than the average plus a standard deviation.

**Figure 7:**
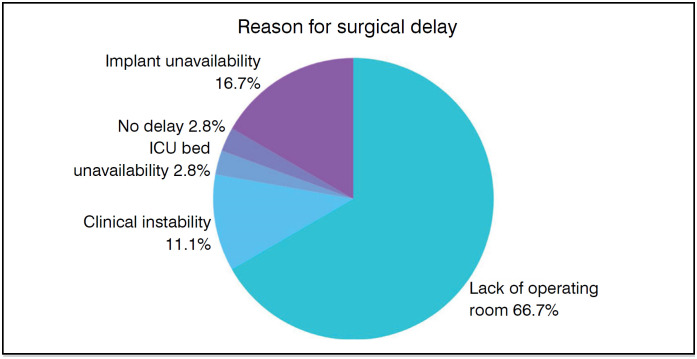
Reason for the surgical delay.

Figure 7 provides information on the reason for the surgical delay, the main one being the availability of a surgical room, present in 24 cases, which is equivalent to 66.7%.

Table 2 presents the results of the statistical analyses and their relevance to this study.

**Table 2:** Results of statistical analysis.

Analyzed variables	Test Used	Results of the tests	Conclusions of the test
Age x Comorbidities	T-Student test	No significant differences were found in the mean age between the comorbidity group (51.2 ± 16.1 years) and the Non-comorbid group (46.6 ± 18.6 years), with a t statistic of −0.161 (p = 0.872).	There is no significant difference in age between groups with and without comorbidities.
Age x Trauma	T-Student test	As t calculated (1.213) is lower than the critical t value (2.306), there is no statistically significant difference in age between the patients who suffered motorcycle trauma and those who suffered car trauma (p = 0.258).	There is no significant difference in age between patients who have experienced different types of trauma.
Age x Complications	Pearson's Correlation	Correlation coefficient approximately −1.6047. Suggest a negative correlation, which means that there is a tendency for complications to decrease with age. However, due to the nature of the complications as categorial or ordinal variables, this interpretation should be done with caution.	Very weak correlation between age and complications. Other factors beyond age can influence complications.
Age x Period of admission for surgery	Pearson's Correlation	The Pearson correlation coefficient between the age of patients and the period of admission for surgery is approximately - 0.6247. This indicates a moderate negative correlation between these variables.	The moderate negative correlation suggests that there is a significant linear relationship between the age of patients and the period of admission for surgery, although other variables and factors may be influencing this relationship. That is, as the age of patients increases, the period of admission for surgery tends to decrease, and vice versa.
Age x Days of hospitalization	Pearson's Correlation	Correlation coefficient approximately 0.4836. This suggests a moderate positive correlation between these variables.	This moderate positive correlation indicates that there is a significant linear relationship between the age of patients and the number of days of hospitalization, although other factors may influence this relationship. That is, as the age of patients increases, the number of days of hospitalization also tends to increase, and vice versa.
Morbidity x Period of admission for surgery	T-Student test	T value of –2.46, showing a significant difference in the periods of admission for surgery between patients with and without morbidities. After calculating the p value, we get approximately 0.025. This means that the p value is lower than the common significance level of 0.05.	There is a significant difference in the period of admission for surgery between patients with and without morbidities.
Morbidity x Complications	T-Student test	T is 0.452. The p value associated with the t-Student test is approximately 0.127, and there is no sufficient statistical evidence to state that there is a significant difference in admissions to surgery between patients with and without complications.	There is no significant correlation between patients with and without complications.
Morbidity x Days of hospitalization	Mann-Whitney U test	The p value of 0.384 indicates that there is no significant difference in hospitalization days between patients with and without morbidity.	The presence of morbidity is not associated with different periods of hospitalization.
Morbidity x Reason for surgical delay	Chi-square test	Qui-square value greater than 20.44 with 4 degrees of freedom. The extremely low p ≈ 1.07e-05 indicates that it is highly unlikely that the association observed between these variables is due to chance, leading us to reject the null hypothesis of independence.	There is a highly significant association between morbidity and surgical delay.
Trauma x Complications	Chi-square test	Chi-square statistics of approximately 0.34 with 4 degrees of freedom. This results in a p-value of approximately 0.99.	There is no significant difference in the presence of complications between the different types of trauma.
Trauma x Period of admission to surgery	Pearson's Correlation	Correlation of −0.12, showing weak and negative correlation between the type of trauma and the period of admission to surgery.	In general, there is a very mild tendency for patients with different types of trauma to have slightly different periods of admission to surgery, but this relationship is not strong enough to be considered statistically significant.
Trauma x Number of hospitalization days	Pearson's Correlation	Correlation of 0.17, indicating that there is a tendency that specific types of trauma are associated with a higher number of hospitalization days. P-value of 0.268.	There is a tendency that specific types of trauma are associated with a higher number of hospitalization days, but the relationship is not very strong. We did not find a statistically significant correlation between the type of trauma and the number of hospitalization days.
Complications x Period of admission for surgery	Pearson's Correlation	Correlation coefficient approximately −0.095 indicates a very weak relationship between the period of admission for surgery and the occurrence of complications. P-value: 0.607.	There is no significant correlation between the complications and the period of admission for surgery. There does not appear to be a clear tendency that a longer period of admission is directly associated with a higher incidence of complications based on the data and statistical analysis performed.
Period of admission for surgery x Reason for the surgical delay	Test F	F ≈ 30.64 and The calculated p-value is approximately 1.37e-10 (i.e. 0.000000000137).	There is evidence that the different reasons for surgical delay are associated with significant differences in the periods of admission for surgery of patients with subaxial fracture.
Complications x Days of hospitalization	Pearson's Correlation	The Pearson correlation coefficient is approximately 0.095 and the associated p-value is approximately 0.685.	There is no significant relationship between complications and the number of days of hospitalization.
Complications x Reason for the surgical delay	Pearson's Correlation	Correlation coefficient approximately −0.175 indicating very weak correlation between these variables. P-value: 0.391.	There is no significant linear relationship between complications and reason for delay in surgery. An weak correlation between complications and reason for the surgical delay means that the presence or absence of complications is not directly related to the reasons that caused the delay in performing the surgery. This may indicate that other factors, besides complications, are contributing significantly to surgical delays, such as availability of resources, hospital protocols, among others.
Hospitalization days x Period of admission for surgery	Pearson's Correlation	Correlation coefficient approximately 0.201, suggesting weak positive correlation between these variables. P-value: 0.261.	There is a slight tendency for patients to spend more time hospitalized as the period until surgery increases. However, this relationship is not statistically significant, indicating only a slight association between these variables.
Days of hospitalization x Reason for the surgical delay	Pearson's Correlation	Correlation of approximately −0.065. p value of 0.689, suggesting a lack of significant correlation between these two variables.	There is no statistically significant association between the period of hospitalization and the reason for the delay in surgery.

## DISCUSSION

The data from this study reveal a significant impact of cervical fractures in the economically active population, with a notably higher prevalence among men. This finding is consistent with previous research, which points to a male predisposition to cervical fractures, possibly related to higher-risk work and recreational activities.^14,15^

The analysis of the age profile of patients revealed that cervical fractures are more common in adults under 50 years of age, with 61.1% of the 36 patients analyzed belonging to this age group. These data corroborate the findings of Gaia et al.^15^ who observed 68.7% of the 264 patients in São Paulo aged between 20 and 50, with car accidents being the main cause of injuries. In addition, the study of Tavares et al.^16^ identified a predominance of male patients in the age group of 20 to 40 years and a lower average age among women. This indicates consistency in the demographic characteristics and risk factors associated with cervical fractures.

Regional variations in trauma mechanisms, such as falls and strokes, indicate that local factors influence the prevalence of cervical fractures. These findings highlight the need for preventive strategies adapted to regional specificities to reduce the incidence of cervical fractures.

Regarding the type and severity of cervical fractures, the use of AO Classification for categorization revealed consistency in the distribution of types A, B and C, despite variations in proportions. These variations can be attributed to the demographic characteristics of the samples and the different trauma patterns observed. Both our study and Gaia et al.^15^ showed a significant proportion of patients with neurological deficits, varying depending on the type of fracture.

The study by Tavares et al.^16^ revealed that approximately 54.1% of patients had neurological deficits during hospitalization, with AO type B fracture, commonly associated with cervical vertebra C5, being the most prevalent.

The study by Khanna et al.^14^ provides a detailed view of the physiological parameters and the preoperative neurological state. The preoperative evaluation using the ASIA scale revealed variations in the patients’ neurological status, reflecting the severity of the spinal cord injuries. Most of the lesions occurred at C5-C6, followed by C4-C5, C6-C7 and C3-C4.

Regarding treatment, the literature emphasizes the importance of surgical intervention for patients with fractal instability and neurological deficits. Khanna et al.^14^ discuss the controversy over the timing of surgical intervention in subaxial traumatic lesions of the cervical spine, a question also addressed in our study. While Khanna et al.^14^ focus on preoperative physiological parameters and at the time of surgery as predictive factors of outcomes, our study highlights that patients with incomplete lesions and stable physiological parameters tend to have better outcomes, regardless of the time of surgery.

The main problems associated with surgical delay include worsening of the injury due to vertebral instability or prolonged spinal cord compression, irreversible neurological damage, and an increased risk of medical complications such as respiratory infections, deep vein thrombosis, and urinary tract infections resulting from prolonged immobilization. In addition, the delay in surgery leads to high costs with hospitalization and rehabilitation. In our study, the costs associated with hospitalization in intensive therapy units (ITI) were analyzed, showing a significant variation between the values of SUS^17^ and SUS Paulista.^18^ Considering an average of R$ 700.00 per day for SUS and R$ 1,500.00 for SUS Paulista, the estimated total cost was R$ 1,162,700,00 and R$ 2,491,500,00, respectively.

These data underline the importance of early interventions to reduce hospital costs and improve the efficiency of the healthcare system.

## CONCLUSION

The results highlight the urgency of addressing the challenges faced by healthcare systems, especially in complex urban contexts such as São Paulo, where efficient management of surgical waiting time can significantly influence clinical outcomes and hospital costs. Implementing strategies to reduce surgical delay will not only promote faster and more effective recovery for patients, but will also optimize the use of hospital resources, reinforcing the continuous need for improvements in the planning and delivery of specialist medical care.

## Appendix 1 Evaluated criteries

1. Gender of the patient
  □ Male
  □ Female
  □ Not specified
2. Age of the patient
3. Comorbidities of the patient
  □ Diabetes
  □ High blood pressure
  □ Heart disease
  □ Obesity
  □ Smoking
  □ Pulmonary disease
  □ Kidney disease
  □ No precedents
  □ Other (specify)
4. Polytraumatized (lesion of 2 or more systems)
  □ Yes
  □ No
5. Trauma mechanism
  □ Auto Accident
  □ Motorcycle Accident
  □ Fall
  □ Dive
  □ Other (specify)
6. Date and time of the trauma
7. Time of entry after the trauma
  □ < 24 hours
  □ 24 – 48 hours
  □ 48 – 72 hours
  □ 72 hours – 7 days
  □ 7 – 30 days
  □ 30 days
8. Level of most severe fracture
  □ C3
  □ C4
  □ C5
  □ C6
  □ C7
  □ C3-C4
  □ C4-C5
  □ C5-C6
  □ C6-C7
  □ C7-T1
9. Number of fractured levels
  □ 1
  □ 2
  □ 3
  □ 4
10. AO Spine Rating (begins with subtype A, B and C)
  □ A0
  □ A1
  □ A2
  □ A3
  □ A4
  □ B1
  □ B2
  □ B3
  □ C
  □ It does not apply. Facet fracture
11. Facet modifier
  □ F1
  □ F2
  □ F3
  □ F4
  □ F5
  □ Not applicable
  □ BL. specify laterality (Example: F4 to the right and F3 to the left = F4,F3)
12. Neurological modifier
  □ N0
  □ N1
  □ N2
  □ N3
  □ N4
  □ NX
13. ASIA
  □ A
  □ B
  □ C
  □ D
  □ E
14. Date and time of surgery
15. Surgical approach
  □ Anterior
  □ Posterior
  □ Combined AP
16. Upper and lower instrumented levels
  □ Anterior route (if applicable) – Upper Level / Lower Level
  □ Posterior route (if applicable) – Upper Level / Lower Level
17. Use of Methylprednisolone
  □ No
  □ Yes (specify dose)
18. Days of hospitalization
19. Reason for the delay between patient entry and the surgical procedure
  □ Clinical instability of the patient
  □ Surgical warning
  □ Availability of implants
  □ Availability of surgical room
  □ Availability of surgical staff
  □ Failure of conservative treatment
  □ Surgeon did not consider as a need for surgical treatment
  □ Unavailability of UTI bed
  □ There was no delay
  □ Other (specify)
20. Complications
21. Use of traction
22. Reduction of fracture
  □ Open
  □ With traction
  □ Both
  □ Other (specify)
